# Mediterranean vineyards and olive groves in Croatia harbour some rare and endemic invertebrates

**DOI:** 10.3897/BDJ.11.e100963

**Published:** 2023-04-20

**Authors:** Barbara Anđelić Dmitrović, Lara Ivanković Tatalović, Tomislav Kos, Petar Crnčan, Domagoj Gajski, Mišel Jelić, Lucija Šerić Jelaska

**Affiliations:** 1 Department of Biology, Faculty of Science, University of Zagreb, Rooseveltov trg 6, Zagreb, Croatia Department of Biology, Faculty of Science, University of Zagreb, Rooseveltov trg 6 Zagreb Croatia; 2 Department for Ecology, Agronomy and Aquaculture, University of Zadar, Trg kneza Višeslava, Zadar, Croatia Department for Ecology, Agronomy and Aquaculture, University of Zadar, Trg kneza Višeslava Zadar Croatia; 3 Croatian Natural History Museum, Demetrova 1, Zagreb, Croatia Croatian Natural History Museum, Demetrova 1 Zagreb Croatia; 4 Department of Botany and Zoology, Faculty of Science, Masaryk University, Kotlarska 2, Brno, Czech Republic Department of Botany and Zoology, Faculty of Science, Masaryk University, Kotlarska 2 Brno Czech Republic; 5 Varaždin City Museum, Šetalište Josipa Jurja Strossmayera 3, Varaždin, Croatia Varaždin City Museum, Šetalište Josipa Jurja Strossmayera 3 Varaždin Croatia

**Keywords:** Araneae, Balkan Penninsula, carabids, endemics, gastropoda, Mediterranean, endangered species

## Abstract

The Mediterranean is characterised by high biodiversity and numerous endemic species. These species are not only present in natural habitats, but also inhabit areas under human influence, such as agricultural lands. In the biodiversity assessment of Mediterranean vineyards and olive orchards within Zadar County, in Croatia, we identified eight endemic species with Mediterranean distribution, six with a Balkan Peninsula distribution, four with Dinaric Alps distribution and three species rare and endangered in Europe. Alongside these species, we have recorded five new species for Croatian fauna, many of those identified by combining morphological characteristics and the DNA barcoding tool. Araneae and Coleoptera contributed the highest number of endemic species and groups with new record were the following: Coleoptera, Diptera and Araneae. Compared to other sites, an olive orchard with ecological pest management (EPM), surrounded by natural ecosystems, had the highest ratio of endemic and rare species. Our findings emphasise that agricultural lands in the Mediterranean can be habitats for endemic and rare species and that future biodiversity research of these habitats is highly important, to monitor potential biodiversity changes and motivate future species and ecosystem conservation.

## Introduction

The coastal part of Croatia, which is situated in the wider Mediterranean region and Balkan Peninsula, has high biodiversity and numerous endemic species (Jelaska et al. 2010). The Mediterranean Region, including the Balkan Peninsula, has high non-marine molluscs diversity and is rich in endemic species (Cuttelod et al. 2011). The Balkan Peninsula and its neighbouring islands are amongst the world's areas with the greatest concentration of terrestrial slugs. There are 101 species of Arionidae, Milacidae, Limacidae and Agriolimacidae in this small area. The vast majority, or 66%, are endemic species with typically relatively small ranges (Wiktor 1997). According to Štamol et al. (2017), there are 328 species and 203 subspecies of land snails inhabiting Croatia. Endemics make a significant part of this malacofauna - 15% of valid species and 46% of valid subspecies are Croatian endemics. Furthermore, both at genus and species level, spiders (Araneae) are another arthropod group that is highly diverse in this area, with numerous endemics within the Balkan Peninsula (Griffiths et al. 2004). Deltshev (1999) identified Croatia as the Balkan Peninsula's country with the second highest number of endemic spider species after Greece. Croatia has 30 endemic spider species, while there are another 31 native to the Balkan Peninsula (Katušić 2017). In terms of insect diversity, Croatia is amongst the richest European countries for Orthopterans (Skejo et al. 2018). Orthopterans are frequently considered significant contributors to farmland biodiversity (Ichihara et al. 2015) and, because the Mediterranean shrubland is especially rich in species, there is a belief that traditional agricultural landscapes have a positive role in keeping the biodiversity of these insects high (Hochkirch 2016). Many orthopteran species and subspecies are present and even recently discovered (Ciplak et al. 2007). Endemic, rare and endangered species of carabid beetles are published within the Red List for Croatia (Vujčić-Karlo et al. 2007) and some of these species are known to appear in the Mediterranean part of the country (Rukavina et al. 2010). Within the genus *Carabus*, there are several endangered and endemic species with a narrow distribution area that encompasses Croatia and neighbouring Bosnia and Hercegovina (Šerić Jelaska et al. 2004, Jambrošić Vladić and Šerić Jelaska 2020, Jambrošić Vladić 2020). For some groups, there is a lack of knowledge on their distribution and ecology which complicates evaluation of endemic species. This applies to the Croatian ant fauna, whose biodiversity is understudied. Considering that part of Croatia is situated in the Mediterranean Region, which has Europe's richest ant fauna, the number of reported ant species is projected to be larger in the Mediterranean part of Croatia in comparison to other parts of the country (Bračko and G 2006). Half of the Croatian ant species have a Mediterranean distribution, i.e. those that are commonly found in the Mediterranean Region (Bračko and G 2006). Species of Diptera and Hemiptera in agricultural lands have wide geographical distribution and are mainly influenced by the crop type (Franin et al. 2021). In this area, wine and olive oil production has a long history and vineyards and olive groves are common parts of the landscape (Froidevaux et al. 2017). Intensive agriculture is considered as a threat to biodiversity alongside other human interventions to the landscape (Ricketts and Imhoff 2003). The main threat of agricultural production to the ecosystem lies in the application of pesticides, mainly insecticides, then chemical fertilisers, with lack of organic matter in soils, but also in the heavy disturbance of the upper soil layers by machinery. Numerous research studies showed negative impacts of pesticide application to the non-target invertebrates in the agricultural ecological systems (Moreby et al. 1997, Pisa et al. 2015, Ivanković Tatalović et al. 2020) with a high negative impact on soil organisms (Gunstone et al. 2021). In this area, wine and olive oil production has a long history and vineyards and olive groves been common parts of the landscape (Froidevaux et al. 2017, Kavvadias and Koubouris 2019). Intensive agriculture is considered as a threat to biodiversity alongside other human interventions to the landscape (Ricketts and Imhoff 2003). The main of them lies in the application of pesticides, mainly insecticides, then chemical fertilisers. According to Barić and Pajač Živković (2020), IPM have wider aims to benefit human and environmental health and to sustain economically balanced agricultural production. The EPM on the other hand is, in the context of agricultural production, an even higher approach in pest management because it reduces to a minimum or excludes the use of synthetic pesticides and fertilisers in general (EC 889/2008), with the aim to benefit agriculture sustainability even more than IPM. Increasing implementation of biological approaches, including biological control, biopesticides, biostimulants and pheromones is a mutually high priority for sustainable agriculture leaders and practitioners, including those working in organic agriculture and IPM (Baker et al. 2020). Research shows that IPM and EPM do not necessarily negatively affect predatory arthropods, carabid beetles and spiders (Bahlai et al. 2010). Šerić Jelaska et al. (2022b) results show that management affects the composition of the carabids community in terms of predator share and functional diversity (relative to control), but there are no significant differences between the two types of management, IPM and EPM.

This paper aimed to identify rare and endemic invertebrate species in agricultural landscapes as a part of biodiversity assay and to analyse the proportion of endemics within vine and olive groves in Zadar County in Croatia. Fauna of rare, endemic and newly-recorded species were presented for the following invertebrate groups: Gastropoda, Araneae (Arahnida) and Insects - Diptera, Coleoptera, Hemiptera, Hymenoptera. The emphasis was given to predator groups Araneae (Arahnida) and Carabidae (Coleoptera, Insecta).

## Materials and methods

### Study sites and sample collection

**Figure 1.**
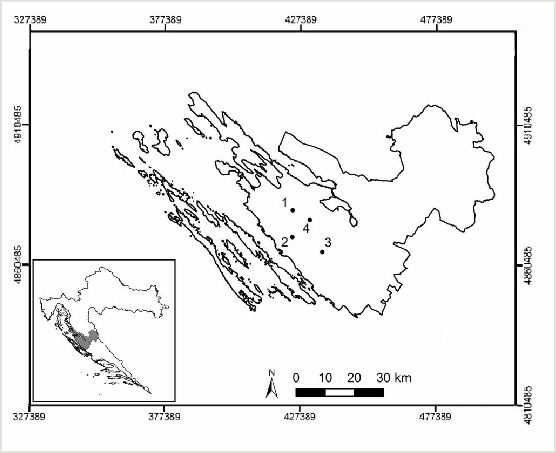
Map of Zadar County, Croatia with four study sites (Transverse Mercator Projection, HTRS96/TM): (1) a vineyard with IPM (located in Baštica); (2) an olive orchard with IPM (located in Škabrnja); (3) a vineyard with EPM (located in Nadin); (4) an olive orchard with EPM (located in Poličnik).

**Figure 2.**
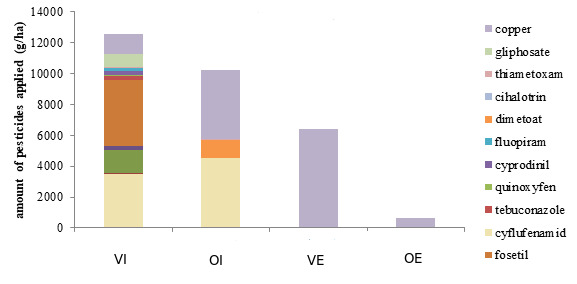
The amount of pesticides added at each study site during 2018, shown as grams of active substances applied per hectare. For site abbreviations, see Table 1.

Studied vineyards and olive groves were situated in Zadar County, in the south of Croatia (Fig. 1). Two sites were within olive orchards, respectively with ecological (OE) and integrated (IO) management and two sites were placed within vineyards with the same management types, ecological (VE) and integrated (VI). Details on agricultural practices applied on these sites are given in Table 1 and Fig. 2. The study locations were in the Köppen climate classification's Mediterranean climatic types (Csa), with wet, mild winters and dry, hot summers (Bolle 2003). Sampling was done in two years, 2018 and 2019, in spring and autumn. The collection periods were in both 2018 and 2019 from April to July and from September to November. Four sampling methods were used: pitfall traps, beating stick with a net method (Schowalter and Chao 2021), Tullgren Funnel method (Macfadyen 1953) and hand picking (Table 2). The first collection method was sampling with pitfall traps (8 cm ø, volume 300 ml) during the season in 2018. Altogether, 12 traps per site were used and placed parallel within the plantation, with the approximate space between the traps of 10 up to 12 m. Traps were buried directly under the olive trees or under the grapevine stumps. Beating stick with a net method was solely applied in vineyards and olive orchards in 2018. The sampling effort was unique during the season and involved hits on five branches on twelve different trees. Sampling of the soil for the Tullgren Funnel method was done once in April and once in October in 2018 at 12 sampling points per site, with 3 litres of the upper 10 cm soil layer being collected.

### Morphological identification and DNA barcoding

Gastropods were identified using the key of Welter-Schultes (2012). Spiders collected by pitfall traps method were classified to species or genus level following araneae - Spiders of Europe site (https://www.araneae.nmbe.ch, accessed on 23 March 2022) (Nentwig 2022). Orthopteran specimens were collected using beating method and pitfall traps and identified using Harz (1969). Carabid beetles collected by pitfall traps were isolated and identified to species following taxonomic keys by Trautner and Geigenmüller (1987), Hurka (1996) and Freude et al. (2004) and the Fauna Europaea nomenclature (Vigna Taglianti 2013). For ant species determination, two keys were used, Seifert (2018) and Lebas et al. (2019) and the AntWeb website (https//:www.antweb.org, accessed on 23 March 2022) (California Academy of Science 2002). For Hemipteran and Dipteran species or morpho-species identification, a photographic guide to Insects of Southern Europe and the Mediterranean was used (Brock 2017).

Species pertaining to the following groups: Araneae and Coleoptera; Carabidae and Chrysomelidae, Hymenoptera; Formicidae, Hemiptera, Diptera, were collected using beating stick with a net method, Tullgren Funnel method and by hand were analysed using the integrative taxonomy approach, combining morphological identification using the above-mentioned taxonomic keys and the DNA barcoding method. Total genomic DNA isolation, PCR amplification, amplicon purification, sequencing and genetic data analysis were performed as described in Anđelić Dmitrović et al. (2022). Individuals identified by the DNA barcoding method are available in the Barcode of Life Database (BOLD) (Ratnasingham and Hebert 2007).

### Data analysis

The information on species distribution was obtained from the following bases: Fauna Europaea (De Jong et al. 2014), Global Biodiversity Information Facility (GBIF) (GBIF org. 2022), MolluscaBase (https://www.molluscabase.org, accessed on 4 February 2022) (MolluscaBase Eds. 2022), araneae - Spiders of Europe (Nentwig 2022), Orthoptera Species File Version 5.0/5.0 (Cigliano et al. 2022), Barcode of Life Data system (BOLD) (Ratnasingham and Hebert 2007, Ratnasingham and Hebert 2013), PESI database (http://www.eu-nomen.eu/ portal/, accessed on 12 May 2022) (de Jong et al. 2015), True hoppers WP database (https://www.truehopperswp.com/, accessed on 12 May 2022), BioLib database (https:// www.biolib.cz/en/main/, accessed on 12 May 2022), The IUCN Red List of Threatened Species (https://www.iucnredlist.org/, accessed on 12 May 2022) and FLOW database (Bourgoin and T 2022). The species was listed as endemic with the Mediterranean, Balkan Peninsula or Dinaric Alps distribution only in cases when all data on distribution were in consent. The list of rare and endangered species, amongst all species recorded at studied sites, was obtained using the Red List in the case of carabid beetles (Vujčić-Karlo et al. 2007), araneae - Spiders of Europe site and communication with experts in the case of spiders. Other groups that contain endemic species were also verified for existence of rare and new species for Croatia through correspondence with taxonomic experts and available data in the above-mentioned databases and current papers (Kuntner 1997, Bračko and G 2006, Borowiec and Salata 2012, Borowiec 2014, Gnezdilov et al. 2014, Skejo et al. 2018, Grbac et al. 2019).

Evenness, as well as Shannon, Simpson and Margalef diversity indices were calculated in PAST 4.03 (Hammer et al. 2001, Happe et al. 2019), for Gastropoda, Araneae and Coleoptera, as these groups were regularly collected across seasons using the pitfall trap method and all specimens pertaining to these groups were identified to species by taxonomic experts.

A Venn diagram, depicting number of species per sampling method, was plotted using online software at https://bioinformatics.psb.ugent.be/webtools/Venn/ (accessed on 25 March 2022).

## Results

### Endemic, rare and species new to Croatia

**Figure 3.**
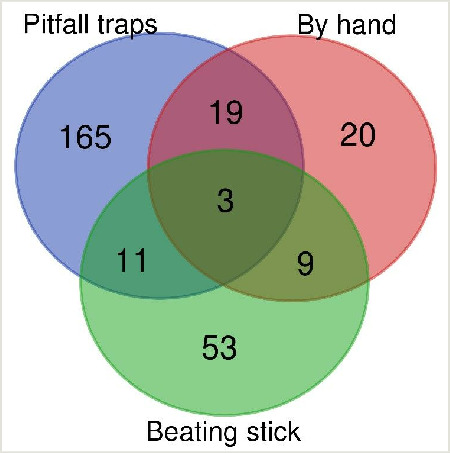
Venn diagram depicting the number of all collected species per sampling method.

Altogether, 280 species of Gastropoda, Araneae, Orthoptera, Coleoptera, Hymenoptera, Hemiptera and Diptera were collected using all sampling methods and the total list of all recorded invertebrate species at five sites is published in GBIF database (Šerić Jelaska et al. 2022a). Pitfall traps yielded most species, followed by the beating stick with a net and hand picking. Only one ant species was collected using the Tullgren funnel (Fig. 3).

Of the invertebrate fauna collected in vineyards and olive orchards in Zadar County, spiders contributed with the highest number of endemic species, six, distributed either in the Mediterranean Region or in the Dinaric Alps (Table 3). In addition, five endemic carabid beetles were collected; three of which are distributed in the Balkan Peninsula and two in the Mediterranean. In the case of Gastropoda species, *Delimasemirugata* is endemic to Croatia and Montenegro and *Tandoniareuleauxi* is endemic to the western Balkan Peninsula and Italy (Bank and Neubert 2020). Additionally, two spider species, *Attuluspenicillatus* and *Zeloteshermani*, are described as very rarely found on the Spiders of Europe site. Orthopterans *Ephippigerdiscoidalis*, *Barbitistesyersini* and *Eupholidopteraschmidti* are endemic to the Balkan Peninsula and Italy. Besides these groups, one hemipteran (*Latissusdilatatus*) and one hymenopteran (*Aphaenogasterpicena*) endemic species with a Mediterranean distribution were recorded in the study area. Furthermore, the collected species *Ditomuscalydonius*, while not considered as an endemic species, is listed on the carabid beetles Red List of Croatia (Vujčić-Karlo et al. 2007) as a critically endangered species. Out of 280 (Šerić Jelaska et al. 2022a) recorded species at our study sites, collected and identified using the above-mentioned methods, five had no previous records for Croatia. Those are, *Ceratinellabrevipes* (Westring, 1851) (Araneae, Lyniphiidae) (Suppl. material 1), one male specimen collected in vineyard with IPM; *Anthomyialiturata* (Robineau-Desvoidy, 1830) (Diptera, Tabanidae) (BOLD:ACE4540) (Suppl. material 2), one specimen recorded in vineyard with IPM; *Corynopteraperpusilla* Winnertz, 1867 (Diptera, Sciaridae) (BOLD:AAN6447) (Suppl. material 3), one specimen recorded in olive orchard with IPM; *Psilopaobscuripes* Loew, 1860 (Diptera, Ephydridae) (BOLD:AAG7016) (Suppl. material 4), 11 specimens recorded in vineyard with IPM; *Aphthonapallida* (Bach, 1856) (Coleoptera, Chrysomelidae) (BOLD:ACZ1493) and one specimen recorded in olive orchard with EPM. The endemic and rare species and those new to Croatia have been listed in the Table 3.

**Figure 4.**
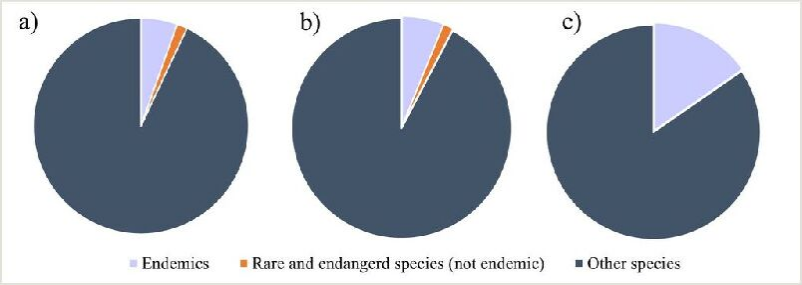
Ratio of endemic and rare to globally distributed spiders (a), carabid beetles (b) and gastropods (c) analysed for all the sites together.

The ratio of endemic, rare and newly-recorded species within Gastropoda, Araneae and Carabidae, comparing to those with wider distribution, is shown in Fig. 4. In the case of Araneae species, endemic and rare species accounted for 7% of the total number of species found. In the Carabidae family, endemics and rare species accounted for 8% of total species, while in Gastropoda, they accounted for 16%.

**Figure 5.**
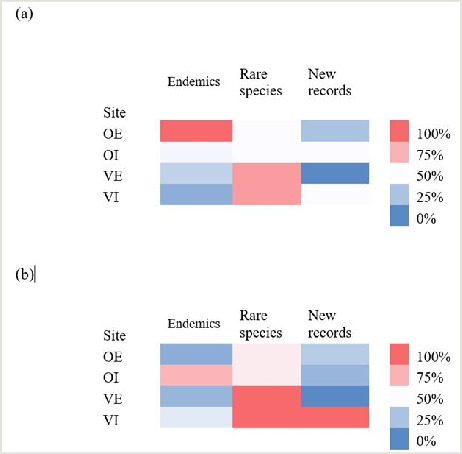
Heat map depicting the ratio of each site in the number of endemic, rare and newly-recorded species (a) and the abundance of the corresponding individuals (b). Study sites are denoted with abbreviations as follows: OE - olive orchard with EPM; OI - olive orchard with IPM; VE - vineyard with EPM; VI - vineyard with IPM.

Comparison of newly-recorded, endemic and rare species distribution amongst our sampling sites showed that, overall, olive orchards with EPM harboured the highest share of endemic species, but not individuals pertaining to these species (Fig. 5). However, the highest abundance and the highest number of newly-recorded species was observed for vineyards with IPM.

### Diversity measures

The Shannon Diversity Index was slightly higher in EPM sites than those with IPM for spiders, but the opposite values were for carabid beetles and gastropods. A similar trend can be observed with Evenness and species richness shown as the Margalef Index, which were higher in IPM than EPM sites. The higest values of diversity indices for Carabidae and Gastropoda was observed in the olive orchard with IPM. Most of the diversity indices measured for Aranaea species were higher in EPM sites. These results are depicted in the Table 4.

## Discussion

The biodiversity of the Balkan Peninsula is still poorly researched, with new species and new records to Croatia being continuously reported (e.g. Francuski et al. (2011), Previšić et al. (2014)). We found no prior records in Croatia for five species noted in this study: one Araneae species, three Diptera species and one Coleoptera species. Our results confirmed that agricultural areas could harbour some rare and endemic species. We also found eight endemics with Mediterranean distribution, six with Balkan Peninsula distribution and four with Dinaric Alps distribution. Despite lower values of measured diversity indices, the total number of endemic species and the abundance of rare species were the highest in olive orchards with EPM. This can probably be explained by the fact that this site is, comparing to the other agricultural sites studied, the only one that is surrounded by natural habitats, which can positively impact invertebrate community in adjacent agroecosystems (Duque-Trujillo et al. 2022). Three species are rare and endangered in Europe. All findings were uploaded in the GBIF database (Šerić Jelaska et al. 2022a) as only the second contribution from Croatia in this global database for faunal research, indicating the need for further biodiversity research of the area, especially having in mind high diversity of fauna and high endemism of this area. Further entries of biodiversity data of this area in the GBIF database and similar databases will enable further meta-analyses of diversity on a larger scale.The following is the list of interesting faunistic records:

**Class Gastropoda**

The Mediterranean Region, from the Iberian Peninsula to the Balkans, is the main centre of diversity and endemism of non-marine molluscs (Cuttelod et al. 2011). Land snails can adapt to challenging environments thanks to a variety of morphological, behavioural and physiological responses to homoeostatic stimuli (Chukwuka et al. 2014). For gastropods, the smallest diversity was observed in the case of olive orchards with EPM. Explanation for this probably lies in the fact that olive orchards with EPM were under more intensive disturbance of soil with mechanisation and this site was characterised by mostly bare soil or with less plant coverage than olive orchards with the IPM site. This negatively impacted gastropod biodiversity in EPM orchards compared to IPM, as most snails and slugs lay their eggs in the ground and need plant coverage for summer aestivation (Godan 1983).

***Delimasemirugata* (Rossmassler, 1836) (Stylommatophora, Clausiliidae)**

*D.semirugata* is a terrestrial gastropod endemic to Croatia and Montenegro, with Croatia containing the type locality (MolluscaBase Eds. 2022). It is a grazer, as is characteristic to the family Clausiliidae (MolluscaBase Eds. 2022). Genus *Delima* is rich in species endemic to Croatia and/or its neighbouring countries on the Balkan Peninsula (MolluscaBase Eds. 2022). *D.semirugata* and its subspecies can be found on the Croatian coast and islands, where they inhabit stony grasslands, smaller rocks and cracks (Štamol et al. 2017). In this study, the species was found on the unmanaged habitat consisting of Mediterranean scrubland, very close to the olive orchard with EPM. Although it was not recorded at the agricultural land itself, this finding proves that the proximity of agricultural activity is not necessarily detrimental to the presence of endemic species.

***Tandoniareuleauxi* (Clessin, 1887) (Stylommatophora Milacidae)**

*T.reuleauxi* is endemic to the Dinaric Alps and can be found in Italy, Slovenia, Croatia, Bosnia and Herzegovina and Montenegro (MolluscaBase Eds. 2022). The species lives in xerophilous forests and can be found crawling on limestone rocks in shaded places. If it is rainy, it is active in broad daylight (De Mattia and Pešic 2015).

**Order Araneae**

Spiders provided the highest number of endemic species in this study because of their capacity to colonise large areas, including agricultural locations (Nyffeler and Sunderland 2003). Despite the high number of endemics with Croatian and Balkan Peninsula distribution (Deltshev 1999), all endemic species were either ones with Mediterranean distribution or Dinaric Alps distribution. This does not necessarily mean that these species are not present in the area. As spiders have a wide range of ecological niches, sampling them requires a combination of techniques and that can leave many species unidentified due to a failure to cover a wide range of microhabitats (Cardoso et al. 2007). Thus, new species and new records are still being described (Deltshev et al. 2022) and this study proved that agroecosystems should not be excluded from such research. Due to their predatory potential on pest species, spiders are essential predatory arthropods in agricultural lands (Samiayyan 2014, Gajski and Pekár 2021). Their richness and diversity promote biocontrol (Cuff et al. 2021). We found that the EPM system resulted in a higher diversity of spiders than the IPM. This is in contrast to prior findings from olive grove studies (Cárdenas et al. 2006, Cardenas et al. 2015). Vineyards with EPM had a higher Shannon and Simpson diversity of spiders than vineyards with IPM, contrary to gastropods and carabid beetles. This could be linked with different ecology and habitat niches between the groups. Spiders are active in canopy and not just on the soil and those species are not directly influenced by soil disturbances with mechanisation, which was more intense in the olive orchard with EPM.

***Attuluspenicillatus* (Simon, 1875) (Araneae, Salticidae)**

There is only ten records of *A.penicillatus* for Croatia, most of them from the late 19^th^ and early 20^th^ century. It is possible that the species is rare in Croatia, but it could also be that it is poorly researched in this part of the country (L. Katušić, personal communication, 15 February 2022). It is described as *very rarely found* on the Spiders of Europe site (Nentwig 2022). This is a globally distributed species that prefers warm places on sandy ground (Nentwig 2022). *A.penicillatus* is an endangered and rare faunistic record.

***Zeloteshermani* (Chyzer, 1897) (Araneae, Gnaphosidae)**

*Z.hermani* is recorded at multiple sites in the Mediterranean Region of Croatia, but always in low numbers (L. Katušić, personal communication, 15 February 2022) and is described as *very rarely found* on the Spiders of Europe site (Nentwig 2022). It has a global distribution and can be found under stones in warm sites (Nentwig 2022). *Z.hermani* is an endangered and rare faunistic record.

***Bassaniodesbufo* (Dufour, 1820) (Araneae, Thomisidae)**

*B.bufo* is a species of Mediterranean distribution (Nentwig 2022) that can be found in olive orchards (Picchi 2020) as was the case in our study, where it was sampled in olive orchards with both EPM and IPM. The earliest record from Croatia is from the second half of the 19^th^ century (Canestrini and Pavesi 1868) and since then, has been recorded at more sites in the Mediterranean Region of Croatia, including the National Park Kornati (Grbac et al. 2019).

***Marinarozelotesholosericeus* (Simon, 1878) (Araneae, Gnaphosidae)**

*M.holosericeus* is a Mediterranean endemic, mostly distributed in the western Mediterranean (Di Franco 1997), but it has been recorded in Croatia (Katušić 2017) and Greece (Nentwig 2022). In our study, it was found in olive orchards with EPM.

***Pulchellodromusbistigma* (Simon, 1870) (Araneae, Philodromidae)**

*P.bistigma* is a small (1.3-1.9 mm) spider with a Mediterranean distribution (Nentwig 2022). The first record for Croatia is from the late 19^th^ century in Dalmatia (Gasperini 1891) and later, it was found in Istria (Muster et al. 2007). We sampled this species in olive orchards, which can be their habitat (Picchi 2020).

***Urocorasmunieri* (Simon, 1880) (Araneae, Agelenidae)**

The area of *U.munieri* is restricted to the region of Dinaric Alps (Venezia Giulia in Italy, Slovenia and Croatia) (Nentwig 2022). The type locality is in Šibenik area, Croatia (Pantini and Isaia 2019). In this research, *U.munieri* was collected at every sampling site with high abundance, indicating that it is well adapted for life on agricultural land.

***Xysticusapricus* L. Koch, 1876 (Araneae, Thomisidae)**

This species was recorded for Croatia for the first time by Drakšić and Katušić (2011) in the National Park Kornati. Before that, it was endemic to Italy (Pantini and Isaia 2019). We found two specimens in the olive orchard with EPM and that is the first recorded for the mainland. It used to be erroneously listed as a Central European species, but now it is considered to be a Mediterranean one (Jantscher 2001).

***Zodarionelegans* (Simon, 1873) (Araneae, Zodariidae)**

*Z.elegans* has a Mediterranean distribution (Nentwig 2022) and was recorded for Croatian by Gasperini (1891). Since then, its presence was noted in Istria, Kvarner, including the Krk Island and Dalmatia Region (Bosmans 1997). We sampled this species in both olive orchards and in vineyards with EPM.

***Ceratinellabrevipes* (Westring, 1851) (Araneae, Linyphiidae)**

*Ceratinellabrevipes* is a species of global distribution and records exist for all Croatia’s neighbouring countries (Komnenov 2010, Nentwig 2022), so its presence was expected. In this study, the species was sampled in olive orchards and vineyards with IPM. This is a new record for Croatian fauna.

**Class Insecta**

Two carabid endemic species from this study with the area limited to the Balkan Peninsula belong to the genus *Carabus*. The Balkan Peninsula is considered a taxon-rich region and the hyper-diverse genus *Carabus* is present in this region with many endemics and endangered species (Šerić Jelaska et al. 2014). In Croatia, thirty species of the genus *Carabus* have been identified, including 53 subspecies (Jambrošić Vladić et al. 2019). Like spiders, carabid beetles are essential predatory arthropods in agricultural lands (Šerić Jelaska et al. 2014, Šerić Jelaska and Symondson 2016), whose richness and diversity promote biocontrol (Šerić Jelaska et al. 2022b). In this study, the IPM system resulted in a higher diversity of carabids than the EPM which is opposite to findings for spiders. The explanation is similar to that in the case of gastropods: Carabids are mainly ground active and juveniles develop in the soil and, thus, could be under the direct influence of soil treatments. This impact of soil disturbances by mechanical methods applied in agricultural sites on the diversity of some groups like carabids has been already confirmed (Kromp 1999).

All three orthopteran endemics belong to the family Tettigoniidae (Bush crickets), which are the largest orthopteran group in Croatia (Skejo et al. 2018). About 20% of bush crickets fauna in Croatia is made of Balkan endemics and stenoendemics, which is due to the physical barriers in the landscape (e.g. Dinaric Alps), variable habitats and for the fact that numerous glacial microrefugia existed in the past (Kenyeres et al. 2009, Skejo et al. 2018). In addition, for many Orthoptera species, their status in the IUCN list is described as data deficient, amongst them *Paramogoplistesnovaki* (Krauss, 1888). The first record of this species was on the island of Hvar (Dalmatia, Croatia) at the close of the 19^th^ century. Since then, several findings of the species were recorded in Croatia as follows: Hvar Island, Neretva River Mouth, Mljet Island and Krka River (Skejo et al. 2018). As a part of our research, *P.novaki* was recorded in an olive orchard with EPM and this represents the most northern record of this species so far and, thus, contributes to the knowledge of the distribution of this rare species. The distribution of this species in Europe, other than localities in Croatia, includes Greece and Italy (Lazio Region and Sardinia) (Hochkirch 2016).

***Ditomuscalydonius* P. Rossi, 1790 (Coleoptera, Carabidae)**

In the case of carabid beetles, notable was the record of the critically-endangered species *D.calydonius* (Vujčić-Karlo et al. 2007). *D.calydonius* needs warms soil to develop (Brandmayr and Brandmayr Zetto 1974). It combines summer aestivation with egg deposition and brood care in the nest, where it collects seeds for larvae to feed on (Brandmayr and Brandmayr Zetto 1974). This record emphasises the fact that agricultural areas could be habitats for rare and endangered species and that it is of high importance to adjust the management type to be more supporting of invertebrate’s diversity (Happe et al. 2019). *D.calydonius* is an endangered and rare faunistic record.

***Carabuscaelatusdalmatinus* Duftschmid, 1812 (Coleoptera, Carabidae)**

*Carabuscaelatus* Fabricius 1801 is a species native to the Alps, Dinarides and western Balkans, with a distribution that spans the Dinaric Mountains (Jambrošić Vladić and Šerić Jelaska 2020). We sampled the subspecies *Carabuscaelatusdalmatinus* in an olive orchard with EPM that is located near the foothills of the Velebit Mountain. *C.caelatusdalmatinus*, common name Dalmatian crimpled ground beetle, is noted in Albania, Croatia and Bosnia and Herzegovina (Löbl and Löbl 2017). The adults are active from May to August (Jambrošić Vladić 2020). It is listed as *Near threatened* in the Red List of Carabid beetles in Croatia, meaning they are not threatened yet, but there is a reasonable concern that they might become in the future (Vujčić-Karlo et al. 2007).

***Carabuscoriaceusdalmaticus* Géhin, 1885 (Coleoptera, Carabidae)**

*C.coriaceus* is widely distributed in Europe (GBIF org. 2022) and subspecies *C.coriaceusdalmaticus* can be found in Croatia, Albania, North Macedonia and Greece (Zicha 2015). It is characterised by smoother elytrae and broader posterior lobes compared to the nominate subspecies *C.coriaceuscoriaceus* (Goidanich 1932). It was collected on all sampling sites.

***Amaradalmatina* Dejean, 1828 (Coleoptera, Carabidae)**

*Amaradalmatina* (Eng. Dalmatian shiny channel runner) is endemic to the Mediterranean Region (Vigna Taglianti 2013). In this study, it was sampled in olive orchards with EPM and these samples, along with their genetic data, are the first entries for this species in the BOLD database (BOLD:AEN2004) (Ratnasingham and Hebert 2013).

***Zabrusincrassatus* (Ahrens, 1814) (Coleoptera, Carabidae)**

This herbivorous carabid species is endemic to the Balkan Peninsula (Vigna Taglianti 2013, Teofilova 2020). In Croatia, it has been sampled at Neretva Delta (Kurbalija 2012) and several localities in Dalmatia (Hvar, Split, Zadar) (Anichtchenko and Guéorguiev 2009). In this study, it was sampled in olive orchards with EPM.

***Olistophusfuscatus* Dejean, 1928 (Coleoptera, Carabidae)**

There are five species of genus *Olistophus* in Europe and they prefer dry habitats on sandy or limy soils (Trautner and Geigenmüller 1987). *O.fuscatus* has a Mediterranean distribution and, while it is not listed in Red List of Carabid beetles in Croatia (Vujčić-Karlo et al. 2007), it is considered to be a rare faunistically (Šerić Jelaska and Temunović 2010). In this study, it was collected in olive orchards with EPM and nearby unmanaged sites.

***Latissusdilatatus* (Fourcroy, 1785) (Hemiptera, Issidae)**

*L.dilatatus* is a type species, by original designation and monotypy, of its genus, which has a Mediterranean distribution (together with Hungary) (Bourgoin and T 2022). It was recorded in Croatia for the first time by Melichar (1906).

***Aphaenogasterpicena* Baroni Urbani, 1971 (Hymenoptera, Formicidae)**

*Aphaenogasterpicena*, endemic to the region of the Dinaric Alps, has only one previous record for Croatia according to www.antweb.org (California Academy of Science 2002, accessed on 21 March 2022). It is widely distributed in Italy, Slovenia and Albania (Boer 2013). This species prefers open fields with little vegetation, like all members of the *Aphaenogaster* group. The first record for this species in Croatia was in Pakoštane, Zadar County (California Academy of Science 2002, accessed on 21 March 2022). In this study, it was recorded in olive orchards with EPM.

***Ephippigerdiscoidalis* Fieber, 1853 (Orthoptera, Tettigoniidae)**

*E.discoidalis* has a distribution range that encompasses zones from Greece to northern Italy (Skejo et al. 2018). In Croatia, it is a common species in a Mediterranean area and it inhabits parts of the Dinaric Alps as well, but only those areaswith a dominant Mediterranean influence (Skejo et al. 2018).

***Eupholidopteraschmidti* (Fieber, 1861) (Orthoptera, Tettigoniidae)**

*E.schmidti* occurs in the Western Balkan Peninsula, from northern Greece in the south, to western Bulgaria, up to northern Italy (Hochkirch 2016, Skejo et al. 2018). It is a medium-sized species, previously considered as a subspecies of *E.chabrieri*, but molecular phylogenetic analysis confirmed its species status (Allegrucci et al. 2014).

***Barbitistesyersini* Brunner von Wattenwyl, 1878 (Orthoptera, Tettigoniidae)**

*B.yersini* is a thermophilic species present in the Mediterranean part of Croatia as well as in southern part of the Dinaric karst (Hochkirch 2016, Skejo et al. 2018). In Europe, it has distribution that includes the Western Balkans and part of central Italy (Hochkirch 2016, Skejo et al. 2018).

***Aphthonapallida* (Bach, 1856) (Coleoptera, Chrysomelidae)**

The Coleopteran species of genus *Aphthona* can be used in biocontrol against weeds, but some species may also cause economic damage on cultivated plants (Özdikmen et al. 2018). The species is widely distributed in Europe (Vigna Taglianti 2013, GBIF org. 2022). In this study, it was recorded in olive orchards with EPM. This is a new record for Croatian fauna.

***Anthomyialiturata* (Robineau-Desvoidy, 1830) (Diptera, Tabanidae)**

The *A.liturata* group is most likely an opportunistic species, with larvae that can develop in a wide variety of organic materials (Suwa and Darvas 1998, Pintilioaie et al. 2021). This species is found throughout Europe; however, its small size makes identification difficult. The first record of this species was obtained in vineyards with IPM using the DNA barcoding method as a part of the MEDITERATRI project (Anđelić Dmitrović et al. 2022). This is a new record for Croatian fauna.

***Corynopteraperpusilla* Winnertz, 1867 (Diptera, Sciaridae)**

Winnertz (1867) established the genus *Corynoptera* for four new species, one of which, *C.perpusilla*, was later chosen as the type species by Enderlein in 1911 (Hippa et al. 2010). Hippa et al. (2010) reported *Corynopteraperpusilla* in Croatia’s neighbouring countries. Species from the genus *Corynoptera* are often examples of the cryptic diversity (Morinière et al. 2019). There is a higher prevalence of unrecorded and ignored species in families with the lowest body sizes, implying that the number of dipteran species in Croatia is likely to be substantially larger than previously reported and, thus, new records are not surprising (Morinière et al. 2019, Anđelić Dmitrović et al. 2022). This genus belongs to the family Sciaridae. Sciaridae, commonly known as Dark wing fungus gnats, are a globally common, but poorly researched dipteran family (Evenhuis et al. 2016), since their small size and superficial homogeneity do not make them attractive to taxonomists and collectors. The first record of this species for Croatian fauna was obtained in olive orchards with IPM using the DNA barcoding method as a part of the MEDITERATRI project (Anđelić Dmitrović et al. 2022). Findings such as this one underline the advantages of molecular tools in species identification, such as the DNA barcoding method (Anđelić Dmitrović et al. 2022, Chimeno et al. 2022).

***Psilopaobscuripes* Loew, 1860 (Diptera, Ephydridae)**

The distribution of this species encompasses European countries: Austria, Bulgaria, the Czech Republic, France, Germany, Greece, Spain and Turkey and North America (Mathis and Zatwarnicki 2010). The first record of this species for Croatian fauna was obtained in a vineyard with IPM using the DNA barcoding method (Anđelić Dmitrović et al. 2022), done as part of the MEDITERATRI project. The flies of this family are often small and this negatively affects their determination.

## Conclusions

We confirmed that endemic and rare species are present in agricultural areas of the Mediterranean part of Croatia supporting the importance of agricultural land in preserving and promoting biodiversity. Rare and endemic species were found under both EPM and IPM management systems, showcasing the positive impact of these closer-to-nature management types. Additional research on regional biodiversity in agricultural landscapes is necessary especially having in mind several endangered species being recorded and agriculture as one of the main drivers for biodiversity decline. Additionally, the first records we had for the area justify the need of further biodiversity assessments, in which agricultural sites should be included.

## Supplementary Material

EBEA1E79-A8D5-590C-AD42-A7F3B0FC0591 10.3897/BDJ.11.e100963.suppl1 Supplementary material 1 Supplementary figure 1 Data type Image Brief description Figure of *Ceratinellabrevipes* (A) dorsal view; (B) dorsal view of pedipalp; (C) ventral view of pedipalp. File: oo_774625.jpg https://binary.pensoft.net/file/774625 MEDITERATRI project team

4FB54F4B-8F85-58EE-ACDC-0C793102E8AB 10.3897/BDJ.11.e100963.suppl2 Supplementary material 2 Supplementary figure 2 Data type Image Brief description Lateral view of *Anthomyaliturata*. File: oo_774626.jpg https://binary.pensoft.net/file/774626 MEDITERATRI project team

FE87705E-4618-5F9F-81D4-A5604864C572 10.3897/BDJ.11.e100963.suppl3 Supplementary material 3 Supplementary figure 3 Data type Image Brief description Lateral view of *Corynopteraperpusilla*. File: oo_774627.jpg https://binary.pensoft.net/file/774627 MEDITERATRI project team

A8F94FFB-3ED2-5E0F-AC22-9F62E9CCCD92 10.3897/BDJ.11.e100963.suppl4 Supplementary material 4 Supplementary figure 4 Data type Image Brief description Lateral view of *Psilopaobscuripes*. File: oo_774628.jpg https://binary.pensoft.net/file/774628 MEDITERATRI project team

## Figures and Tables

**Table 1.** The list of study sites with additional information on location of the site, abbreviation, pest management type applied on the site, size of the area, vegetation and weed processing and soil processing in the terms of mulching, ploughing and number of pesticides treatment. Added pesticides have been grouped according to the main active compounds, those being synthetic (Organochlorides and chlorinated hydrocarbons, Organophosphates, Pyrethroids, Neonicotinoids and Ryanoids), biological (Bt kurstaki, Spinosad) and copper ones (Copper(I) oxide or copper oxychloride).

Type of Study Site	Location	Abbreviation	Pest Management Type	Area (ha)	Vegetation and weed procession	Mulching	Ploughing	Synthetic pesticides*	Biological pesticides**	Copper compounds
Olive orchard	Poličnik, Zadar County, Croatia	OE	ecological	24	Rocky soil with little plant coverage and regular mowing	Yes	No	0	4	5
	Škabrnja, Zadar County, Croatia	OI	integrated	0,85	Grass coverage, surrounded by coppice, and regular mowing	Yes	No	6	0	3
Vineyard	Nadin, Zadar County, Croatia	VE	ecological	15	Tilled soil with weeds	Yes	Yes	0	0	6
	Baštica, Zadar County, Croatia	VI	integrated	6	Tilled soil with weeds	Yes	Yes	12	0	3

**Table 2.** Total number of identified species per taxonomic group and per sampling method used. Number zero (0) indicates that no specimens were caught by the method, while dash (/) indicates that specimens were caught, but not identified to the species level.

**Taxon**	**Number of species per sampling method**			
	Pitfall traps	Beating stick	By hand	Tullgren funel
**Gastropoda**	11	/	/	0
**Araneae**	116	30	7	0
**Orthoptera**	/	11	0	0
**Carabidae**	65	0	24	0
**Other Coleoptera**	/	0	8	0
**Hymenoptera**	5	11	3	1
**Hemiptera**	/	15	7	0
**Diptera**	/	14	2	0

**Table 3.** List of endemic, rare and endangered species collected in all four sampling sites. M – species with the area limited to the Mediterranean Region; D – species with the area limited to the region of the Dinaric Alps; B – species with the area limited to the Balkan Peninsula.

**Class**	**Order**	**Family**	**Species**	**Endemics**	**Rare and/or endangered species**	**New records for Croatia**
**Gastropoda**	Stylommatophora	Clausiliidae	*Delimasemirugata* (Rossmassler, 1836)	B		
	Stylommatophora	Milacidae	*Tandoniareuleauxi* (Clessin, 1887)	B		
**Arachnida**	Araneae	Thomisidae	*Bassaniodesbufo* (Dufour, 1820)	M		
	Araneae	Salticidae	*Attuluspenicillatus* (Simon, 1875)		*	
	Araneae	Lyniphiidae	*Ceratinellabrevipes* (Westring, 1851)			*
	Araneae	Gnaphosidae	*Zeloteshermani* (Chyzer, 1897)		*	
	Araneae	Gnaphosidae	*Marinarozelotesholosericeus* (Simon, 1878)	M		
	Araneae	Philodromidae	*Pulchellodromusbistigma* (Simon, 1870)	M		
	Araneae	Agelenidae	*Urocorasmunieri* (Simon, 1880)	D		
	Araneae	Thomisidae	*Xysticusapricus* L. Koch, 1876	D		
	Araneae	Zodariidae	*Zodarionelegans* (Simon, 1873)	M		
**Insecta**	Diptera	Tabanidae	*Anthomyialiturata* (Robineau-Desvoidy, 1830)			*
	Diptera	Sciaridae	*Corynopteraperpusilla* Winnertz, 1867			*
	Diptera	Ephydridae	*Psilopaobscuripes* Loew, 1860			*
	Coleoptera	Carabidae	*Carabuscaelatusdalmatinus* Duftschmid, 1812	B		
	Coleoptera	Chrysomelidae	*Aphthonapallida* (Bach, 1856)			*
	Coleoptera	Carabidae	*Carabuscoriaceusdalmaticus* Géhin, 1885	B		
	Coleoptera	Carabidae	*Zabrusincrassatus* (Ahrens, 1814)	B		
	Coleoptera	Carabidae	*Amaradalmatina* Dejean, 1828	M		
	Coleoptera	Carabidae	*Ditomuscalydonius* P. Rossi, 1790		*	
	Coleoptera	Carabidae	*Olisthopusfuscatus* Dejean, 1828	M		
	Hemiptera	Issidae	*Latissusdilatatus* (Fourcroy, 1785)	M		
	Hymenoptera	Formicidae	*Aphaenogasterpicena* Baroni Urbani, 1971	D		
	Orthoptera	Tettigoniidae	*Ephippigerdiscoidalis* Fieber, 1853	D		
	Orthoptera	Tettigoniidae	*Eupholidopteraschmidti* (Fieber, 1861)	B		
	Orthoptera	Tettigoniidae	*Barbitistesyersini* Brunner von Wattenwyl, 1878	M		

**Table 4.** Diversity indices for Araneae, Carabidae and Gastropoda collected by pitfall traps for each research site.

**Site**	**Taxon**	**Shannon H**	**Simpson 1-D**	**Evenness e^H/S**	**Margalef**
Olive orchard with EPM	Gastropoda	0.01	0	0.51	0.15
	Araneae	3.54	0.96	0.63	9.87
	Carabidae	1.87	0.71	0.27	4.22
Olive orchard with IPM	Gastropoda	1.41	0.69	0.68	0.98
	Araneae	3.29	0.93	0.33	11.72
	Carabidae	2.3	0.86	0.3	4.47
Vineyard with EPM	Gastropoda	1.26	0.8	1.17	1.24
	Araneae	3.53	0.95	0.69	9.27
	Carabidae	2.02	0.8	0.24	3.93
Vineyard with IPM	Gastropoda	1.01	0.44	0.46	1.41
	Araneae	3.21	0.91	0.41	9.8
	Carabidae	2.14	0.83	0.28	4.42
