# Supplementary figures and images for: Mediterranean vineyards and olive groves in Croatia harbour some rare and endemic invertebrates
Source: Biodivers Data J. 2023 Apr 20;11:e100963. doi: 10.3897/BDJ.11.e100963 (PMC10850877; doi:10.3897/BDJ.11.e100963)

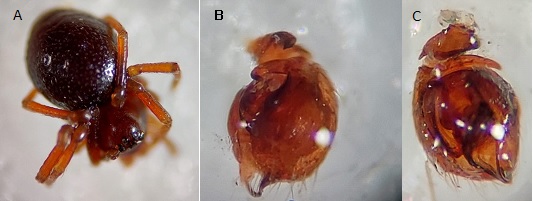

Supplement: Supplementary material 1 — Supplementary figure 1 [file bdj-11-e100963-s001.jpg]

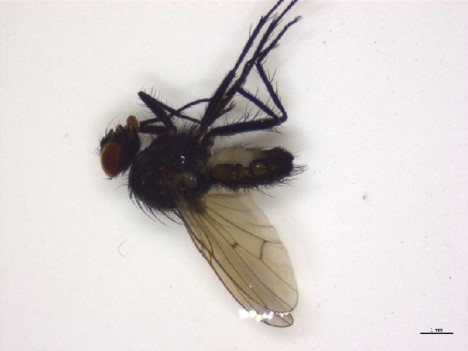

Supplement: Supplementary material 2 — Supplementary figure 2 [file bdj-11-e100963-s002.jpg]

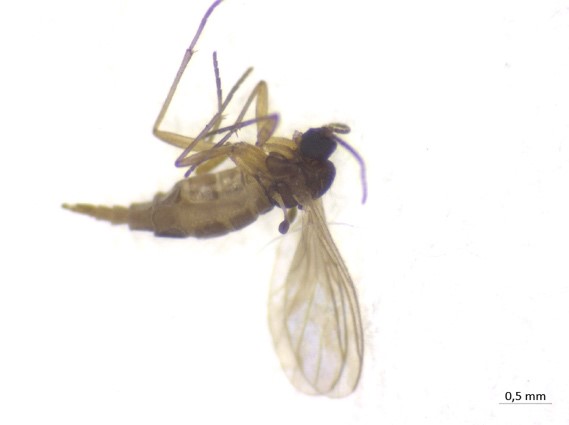

Supplement: Supplementary material 3 — Supplementary figure 3 [file bdj-11-e100963-s003.jpg]

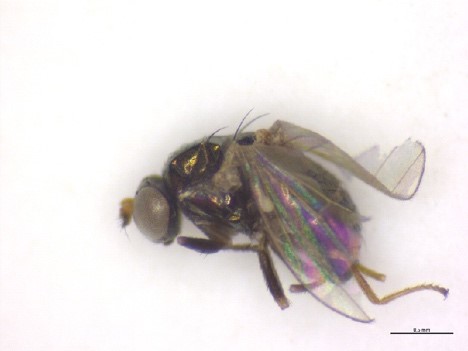

Supplement: Supplementary material 4 — Supplementary figure 4 [file bdj-11-e100963-s004.jpg]
